# Extra‐cardiac vagal stimulation: Clinical utility of a novel diagnostic and therapeutic tool in supraventricular tachycardia

**DOI:** 10.1002/joa3.70134

**Published:** 2025-07-15

**Authors:** Jose Carlos Pachon‐M, Enrique Pachon‐M, Tasso Lobo, Tomas Santillana‐P, Carlos Pachon, Juan Pachon‐M, Christian Higuti, Maria Zelia Pachon, John Clark

**Affiliations:** ^1^ São Paulo University São Paulo Brazil; ^2^ São Paulo Heart Hospital São Paulo Brazil; ^3^ Akron Children's Hospital Akron Ohio USA

**Keywords:** ablation, accessory pathway, Cardioneuroablation, supraventricular tachycardia, Wolff‐Parkinson‐White

## Abstract

**Background:**

The differential diagnosis of supraventricular tachycardias (SVTs) is essential during radiofrequency‐(RF) ablation. The extracardiac vagal stimulation (ECVS), introduced in 2015, offers new insights for electrophysiological studies and ablation, allowing controlled cardiac vagal effect.

**Methods:**

Prospective study of 625 SVT ablation patients. ECVS was performed using a regular electrophysiology catheter to study atrioventricular (AV) and ventriculo‐atrial (VA) conduction and their effects on tachycardia. Baseline ECVS was performed to determine the optimal position for right or left ECVS, near the jugular foramen. ECVS was repeated during atrial and ventricular pacing (VP) to monitor the procedure's progression and ensure successful endpoints.

**Results:**

ECVS was successful in 611/625 patients (98%), 381 (62.3%) had AV node reentry tachycardia‐(AVNRT), and 230 (37.6%) accessory pathway (AP), including 135‐(58.7%) anterograde AP (WPW) and 95 (41.3%) concealed AP. ECVS + VP in 33 patients with atypical AVNRT yielded VA block in 32‐(97%), suggesting VA conduction solely via the AV node. In contrast, 57 patients with concealed para‐septal AP maintained VA conduction during ECVS, confirming AP. ECVS proved to be a fast, reliable, and practical additional EP tool: VA block indicated AVNRT, while persistent VA conduction suggested AP. Additionally, ECVS was highly effective in revealing and confirming successful AP ablation by demonstrating the absence of AV and VA anomalous conduction.

**Conclusion:**

ECVS was a valuable tool in the diagnosis and ablation of SVTs. It allowed reproducible AV and VA block through normal pathways, easily identifying AVNRT and concealed, intermittent, or subtle AP. It was particularly useful in complex cases involving concealed AP and atypical AVNRT tachycardia.

## Graphical abstract

Extracardiac vagal stimulation (ECVS) is a novel, safe tool inducing controlled AV/VA block. It assists SVT differential diagnosis, pathway unmasking, and ablation endpoint confirmation, adding value to EP procedures for supraventricular tachycardias.

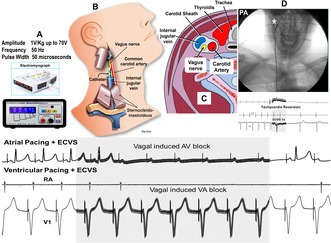

## 1 INTRODUCTION

This study is a contribution to the differential diagnosis of SVTs and RF ablation,^1^, ^2^ using neurostimulation. Parasympathetic effects, such as those elicited by the Valsalva maneuver^3^ and carotid sinus massage, have long been utilized as diagnostic and therapeutic tools.^4^, ^5^ In this context, in addition to saving time, ECVS represents a promising novel approach that could enhance the accuracy and efficacy of SVT EP studies and ablation, offering a new option at the operator's discretion.

In 2015, extra‐cardiac vagal stimulation (ECVS)^6^ was introduced, aimed at achieving a rational control and endpoint for CNA^7^ in the treatment of vasovagal syncope, functional bradyarrhythmias, and AF. It allows for the controlled reproduction of vagal effects. Inhibition of the AV node causes immediate transitory AV and VA block of normal pathways, ruling out an AP.

During EP procedures for SVT, diagnosing mechanisms such as atrial tachycardia and AVNRT versus AVRT often relies on complex and time‐consuming maneuvers.^8^, ^9^, ^10^ This is particularly true for concealed para‐septal APs^11^, ^12^ and atypical AV node reentry cases.^13^, ^14^ Additionally, at the onset of manifest AP procedures, such as those for WPW, it is beneficial to maximize the preexcitation pattern.^15^ As an endpoint, inducing transient AV and/or VA block is essential to confirm the successful elimination of the AP and to reveal any additional subtle AP.^16^

In this context, ECVS may serve as an adjunct tool in the diagnosis and ablation of SVT, contributing to differential diagnosis during complex EP procedures.

## 2 METHODS

**FIGURE 1:**
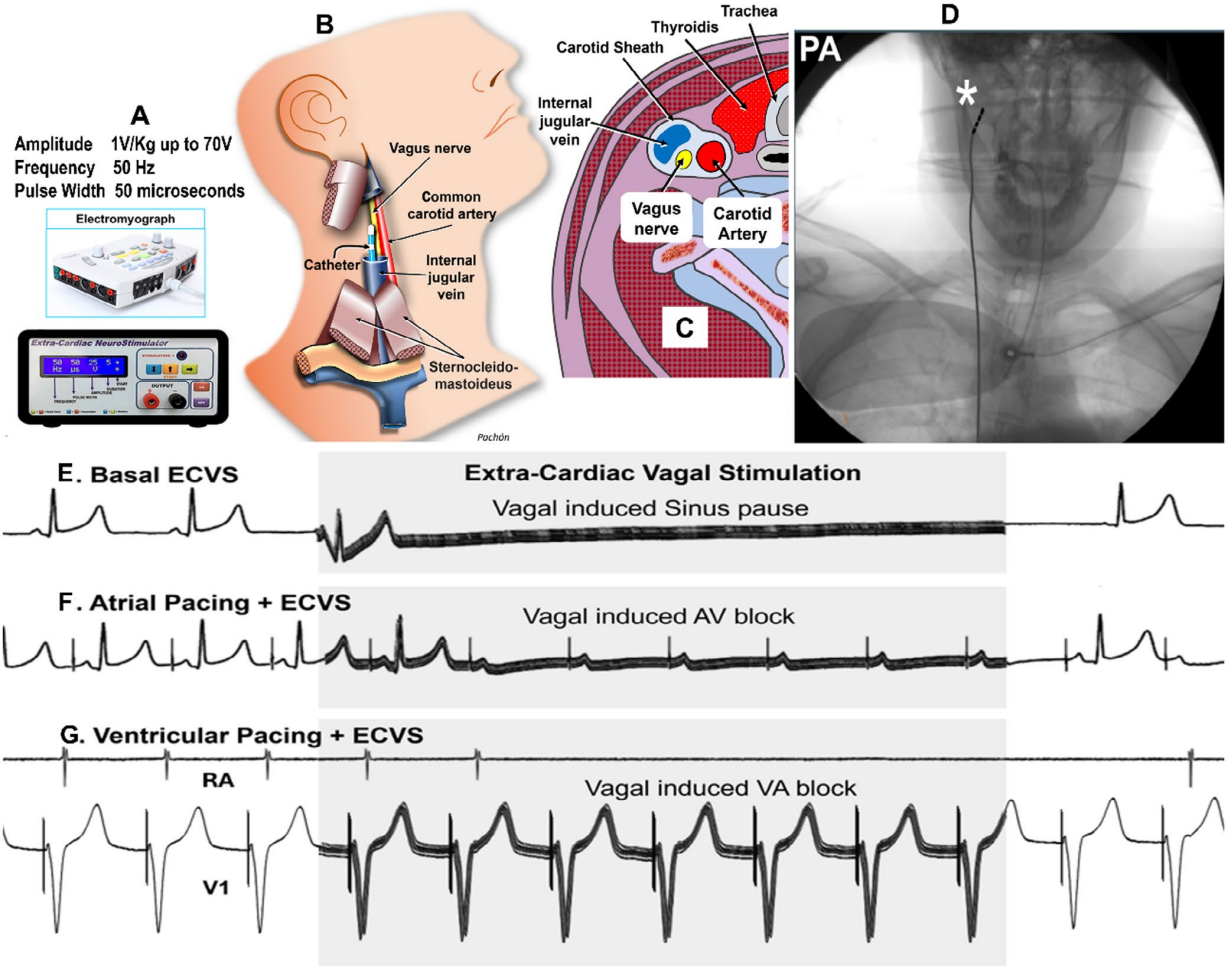
Illustration and effects of extracardiac vagal stimulation (ECVS). (A) Typical parameters of the ECVS, including pulse duration, frequency, and amplitude (B) Schematic of the ECVS procedure showing the catheter inside the internal jugular vein, without direct contact with the vagus nerve. (C) Transverse section of the neck illustrating the proximity between the internal jugular vein and the vagus nerve. (D) x‐ray image of the ECVS catheter in the posteroanterior (PA) view. (E) Normal effect of ECVS on the sinus node, evidenced by a sinus pause. (F) Effect of ECVS on AV conduction during atrial pacing, showing a transient vagal‐induced AV block. This is normal behavior, indicating no abnormal AV pathways. (G) Effect of ECVS on VA conduction during ventricular pacing, with the induction of transient VA block. This normal response helps rule out abnormal VA pathways.

Prospective study including 625 patients with standard indications for SVT ablation (AVNRT, AVRT with, and without preexcitation) according to current guidelines, from January 2015 through June 2020. ECVS was attempted in all patients using an additional regular EP catheter to study AV and VA conduction (Figure 1F,G) and their effects on tachycardia. This catheter was kept in this position throughout the procedure to facilitate further stimulations. Any drugs that could interfere with the autonomic nervous system were suspended, and cases where this was not possible were excluded.

**FIGURE 2:**
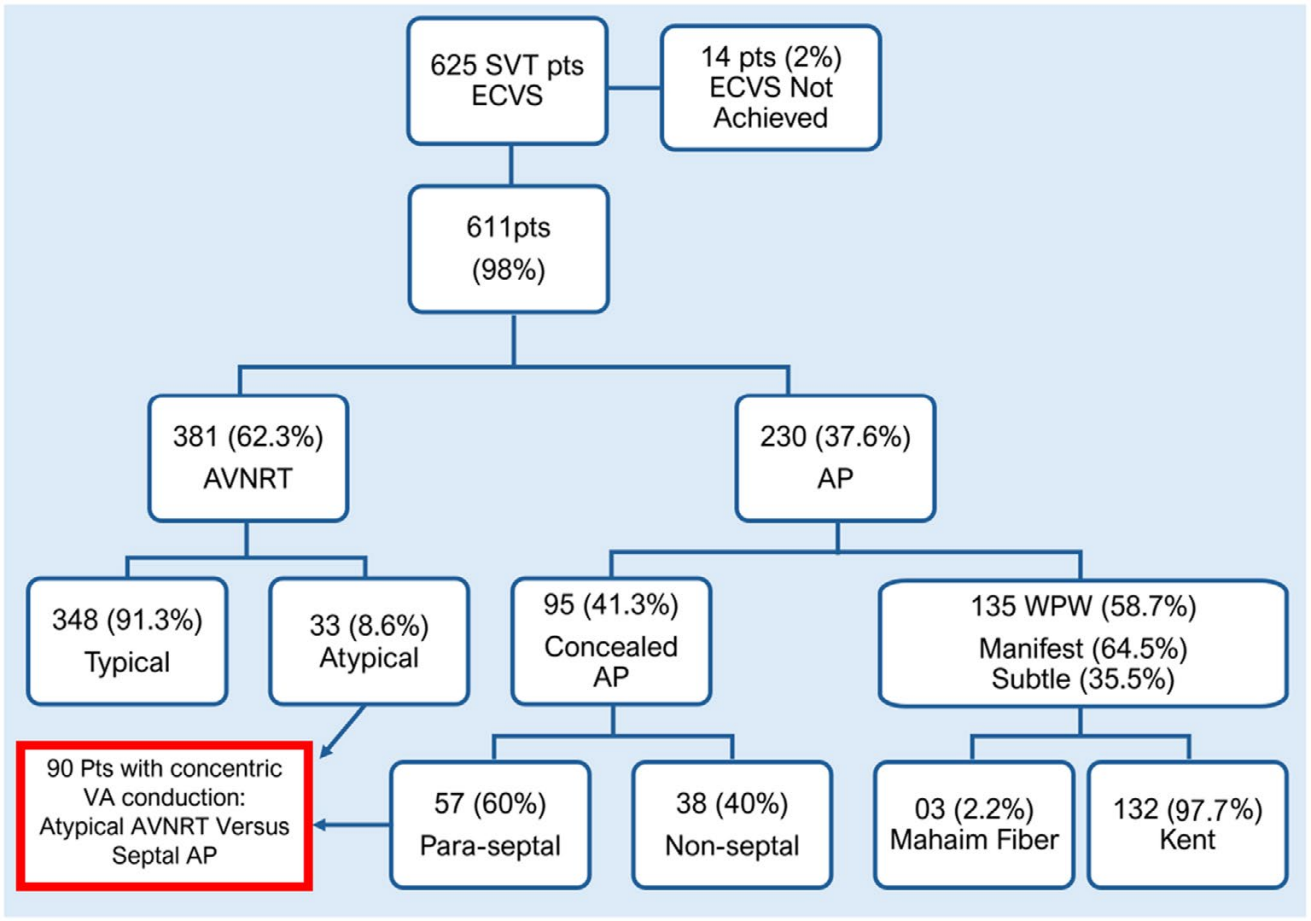
This flowchart illustrates the distribution of patients of this study. Six hundred twenty‐five cases were initially considered. ECVS was successful in 611 (98%) patients, and it was not achieved in 14 patients (2%). Special Focus: 90 patients with concentric VA conduction were further analyzed with ECVS to distinguish between atypical AVNRT and septal AP. Para‐septal AP was found in 57 cases. This detailed breakdown highlights the distribution of patients across different types of SVT and the success rate of ECVS, providing an overview of the study's scope and focused areas. The red box indicates the group for which ECVS has the highest differential diagnostic value.

Initial ECVS^6^ was performed as an additional EP test to establish a baseline vagal effect assessment, Figures 1A,B,E–G and 2. It was also repeated during the ablation to monitor the progression and a successful endpoint. The first ECVS was conducted to determine the optimal position for right vagal stimulation, typically near the jugular foramen just above the root of the wisdom tooth, Figure 1B–D, to elicit the most significant vagal response, Figure 1E.

**FIGURE 3:**
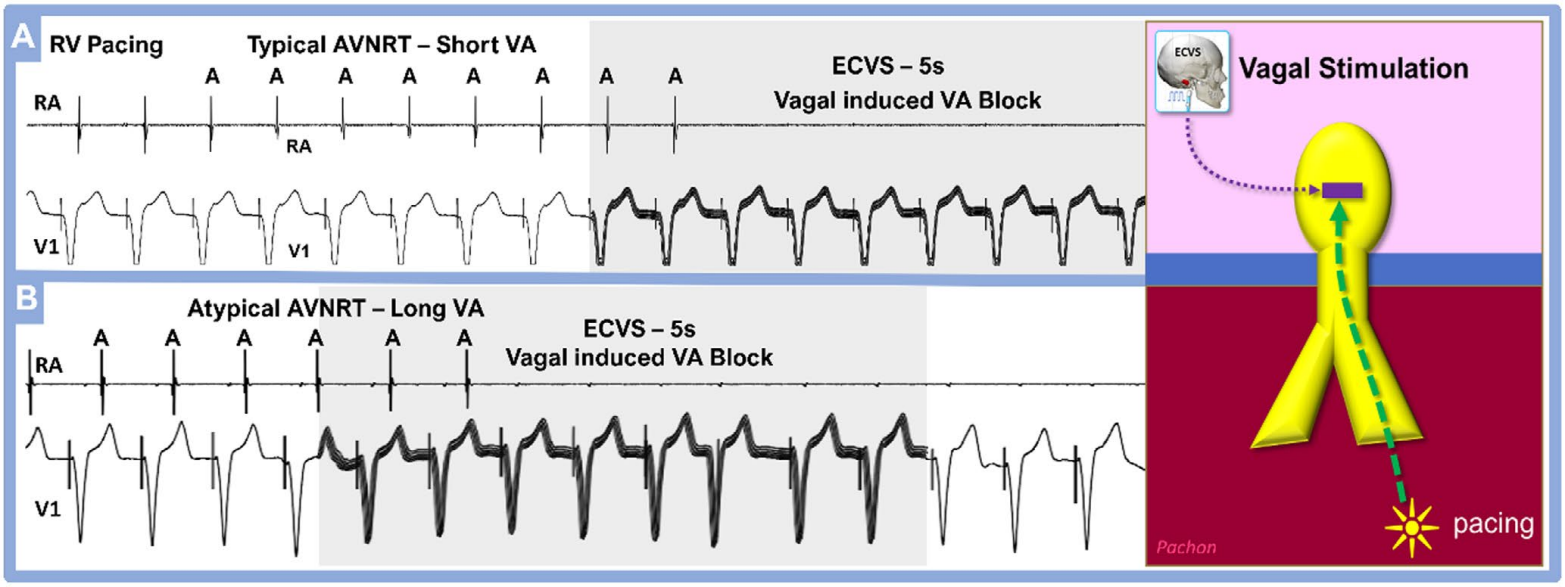
(A) Typical AVNRT (short VA) and (B) Atypical ANVRT (long VA) patients preablation. The ECVS typically induces VA block both before and after ablation.

**FIGURE 4:**
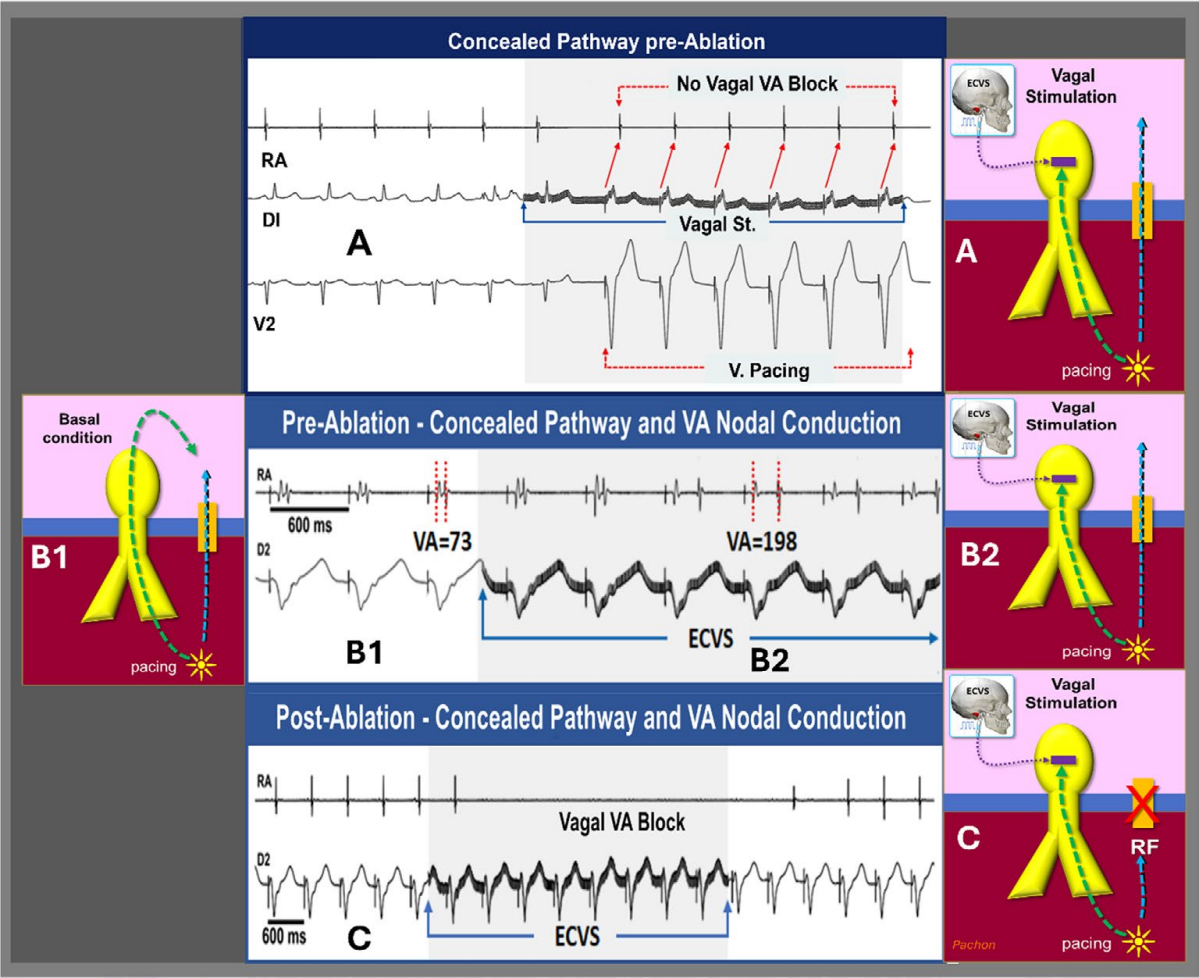
ECVS in AVRT. (A) Persistent VA conduction during ECVS with VP suggests the presence of AP. (B) ECVS showing dual VA conduction; (B1) By AV node and AP before the ECVS; (B2) Under VP the ECVS changed the VA conduction pattern, with the VA interval suddenly increasing from 73 ms to 198 ms. This change occurred because of sudden VA nodal conduction block, maintaining VA conduction through the AP only. (C) After AP ablation, there is normal VA conduction through the AV node during VP. However, during ECVS, there is an immediate vagal‐induced VA block, indicating the absence of ectopic VA conduction and confirming the successful AP ablation.

Subsequently, ECVS was repeated during right atrial pacing to study AV conduction, Figure 1F. Again, ECVS was performed during VP to study VA conduction, Figures 1G, 3 and 4. If no AV or VA block occurred during ECVS, the procedure was repeated with the stimulation catheter positioned on the left vagus. The tests were reproduced throughout the procedure as deemed appropriate by the operator, and at the end to compare with the baseline condition. A quadripolar catheter was placed and kept in the internal jugular vein to facilitate further vagal stimulations as needed.

ECVS information was compared with standard electrophysiological maneuvers (conduction time, atrial activation sequence during tachycardia, para‐Hisian pacing, postpacing interval during tachycardia, and His synchronous ventricular extrastimulus during SVT), Figures 3 and 4. Most of the technical aspects of the ECVS were previously published^6^; however, the main points are discussed below.

## 3 ETHICS DECLARATION

This study was based on our regular clinical application of diagnosing and ablation for patients routinely treated in our hospital. After a detailed in‐person interview with a thorough explanation of the study's objectives, procedures, potential risks, and benefits, each patient provided written informed consent prior to treatment. The study was conducted in accordance with the ethical principles outlined in the Declaration of Helsinki, ensuring the protection of the rights, safety, and well‐being of the participants. The protocol was approved by the ethics committees and is fully concordant with all regulations of our country. Robust measures were implemented to safeguard the data collected during the study, in compliance with local regulatory requirements and international ethical standards.

### 3.1 Vagal stimulator

The ECVS was performed according to the technique previously published, Figure 1A, by using DC stimulation with square wave pulses of 50 microseconds duration, a frequency of 30 to 50 Hz, and an amplitude from 10 to 70 V, adjusted according to patient weight^6^; however, any generic neurostimulator capable of these settings can be used. An extremely short pulse duration, amplitude, and limitation of current, duty cycle, and specific output impedance protection were included to prevent tissue damage. Additionally, the system allows pulse delivery only within an acceptable impedance window. A timer function also enabled the application of pulse trains with predefined timing, usually 1/2 to 5 s. However, the ECVS may be performed with any neurological stimulator able to be adjusted with the specific parameters.

### 3.2 Extracardiac vagal stimulation (ECVS)

Most of ECVS (92%) was performed from the right internal jugular vein, Figure 1A.^6^ Vascular access was the right femoral vein. The stimulation was obtained by an endovascular electrical field from the internal jugular vein between the distal and third pole of a regular quadripolar EP catheter, Figure 1B. In some cases, because of ease of handling and operator preference, the RF catheter, adequately disconnected from the RF generator, was occasionally used for ECVS. There was no direct contact with the vagus nerve. As the distance between the nerve and the catheter in the internal jugular vein may vary, Figure 1C, an amplitude of 1 V/kg, limited to 70 V, was used, with a remarkably short pulse width of 50 microseconds and a frequency of 50 Hz for 1/2 to 5 s, Figure 1A,E–G. The best stimulation is obtained at the level of the jugular foramen, with the catheter tip turned medially, Figure 1D. In cases with difficult right vagus access, the left vagus was stimulated (8%), or even any vagus nerve at the neck level, guiding the best point by ultrasound. The transducer was placed transversely to visualize the vascular‐nervous bundle, and the catheter was positioned endovascularly at the level where the vagus nerve was seen closest to the internal jugular vein. The total time to achieve the first ECVS was evaluated.

As per protocol in our service, all cases were performed with general anesthesia under endotracheal intubation and BIS index control between 35 and 40 to ensure patient comfort, safety, and autonomic stability. However, the procedure may be performed under sedation. The anesthesia team was instructed to avoid any drugs, such as atropine, catecholamines, rivastigmine, and so forth, that could modify the parasympathetic or sympathetic tone.

## 4 STATISTICAL ANALYSIS

The distribution of continuous variables was evaluated using the Kolmogorov–Smirnov (K‐S) test. Results for continuous variables are reported as mean ± standard deviation (SD) for normally distributed data, and median with interquartile ranges (IQR) for data not normally distributed, analyzed with nonparametric tests. For continuous variables that assumed a normal distribution but potentially had unequal variances, robust Welch's t‐test was employed, which adjusts for such differences. The Mann–Whitney U test, a nonparametric test, was used to compare medians of variables that were not normally distributed. Statistical significance was set at a two‐sided *p*‐value of less than 0.05, with appropriate adjustments made for multiple comparisons. Data analysis was conducted using SPSS (version 28.0.1.1) and the latest version of Jamovi (2023, Version 2.4.14.0).

## 5 RESULTS

In total, 625 patients underwent ECVS and ablation of SVT, Figure 2. The mean age was 40.2 ± 17 years, with 368 women (59%) and 257 men (41%) with no significant cardiopathy (Ejection Fraction: 0.68 ± 0.07), Table 1.

**TABLE 1:** Summary of patient distribution and ECVS outcomes in SVT ablation study.

Case description	Number of patients	Percentage/statistic
Patients undergoing ECVS and SVT ablation	625	100%
Age 40.2 ± 17 years		
Gender		
Women	368	59%
men	257	41%
Ejection fraction	625	68 ± 7%
Diagnosis		
AV node reentry tachycardia (AVNRT)	**381/625**	**62.30%**
Typical AVNRT	348/381	91.3%
Atypical AVNRT (aAVNRT)	33/381	8.6% of AVNRT
PJRT (Coumel)	3/33	9.1% of aAVNRT
AP	**230**	**37.60%**
WPW	135/230	58.7% of AP
Manifest (stable)	87/135	64.5%
Subtle	48/135	35.5%
Mahaim	3/135	2.2%
Concealed AP	95/230	41.3% of AP
Concealed para‐septal AP	57/97	58.8% of concealed AP
AP localizations		
Left lateral AP	109/230	47.4% of AP
Posteroseptal AP	59/230	25.7% of AP
Right septal AP	37/230	16.1% of AP
Left septal AP	22/230	9.6% of AP
Right free wall AP	13/230	5.7% of AP
Anteroseptal AP	7/230	3.1% of AP
Multiple APs	17/230	7.4% of AP
ECVS		
ECVS successful	611/625	97.8%
ECVS not achieved	14/625	2.2%
Jugular vein access for ECVS		
Right jugular vein	562/611	91.9%
Left jugular vein	49/611	8.1%
Time to optimal jugular spot		
Right jugular vein		4 ± 2 min
Left jugular vein		7 ± 3 min
ECVS procedures		
Total ECVSs performed		3380
Mean ECVSs per patient		5.2 ± 4
Baseline ECVS test pause		8 ± 4 s
Heart rate increase postpause		9 ± 5 bpm

*Note*: This table provides a detailed overview of the patient distribution and outcomes of ECVS in the study of SVTs (SVTs). It highlights the total number of patients, the success rate of ECVS, and the distribution of specific SVT types, including atypical AV node reentry tachycardia (aAVNRT) and accessory pathways (APs). The table also indicates the proportion of patients with manifest and concealed APs, as well as the response to ECVS in terms of VA block and maintenance of VA conduction. Additionally, the table breaks down the localization of APs, providing a comprehensive view of the anatomical distribution in the study population.

ECVS was successful in 611 patients (97.7%). In 14 cases (2.3%), ECVS was not possible because of impossible right and left jugular vein catheterization in 8 (1.3%) cases and a bad site for vagal stimulation in 6 (0.96%) cases.

The 611 patients (98%) with full planned ECVS constitute the analysis group for this study; 381 (62.3%) had AVNRT, and 230 (37.6%) had AP, 135 (58.7%) being manifest AP (WPW), and 95 (41.3%) concealed AP, Figure 2.

Among the 381 patients with AV node reentry tachycardia, 348 had a typical (91.3%) and 33 had atypical AVNRT (8.7%), Figure 3.

In 562 patients (91.9%), the ECVS was performed in the right, and in 49 (8.1%), it was performed in the left jugular vein. The catheter handling time to the optimal jugular site was 4 ± 2 min in the right and 7 ± 3 min in the left jugular vein. This time was compensated by the reduced need for multiple diagnostic maneuvers. Per protocol, the first attempt was the right vagus. The left was accessed only if the right was not possible. A total of 3380 ECVSs were performed, with a mean of 5.2 ± 4 ECVS per patient. Three sets of ECVS tests were conducted before ablation.

The first (baseline test) produced a mean pause of 8 ± 4 s, usually because of sinus arrest (Figure 1E), but sometimes because of a sinus pause with a sinus escape not conducted to the ventricles (AV block). Usually, sinus rhythm recovers after the pause with a heart rate on average 9 ± 5 bpm faster than the initial heart rate.

In the second test, ECVS during atrial stimulation produced second, third, or high‐degree AV block in all cases of AVNRT (381 patients), concealed AP (95 patients), or Coumel cases, (Table 2 and Figure 1F). In patients with manifest WPW, ECVS led to an increase in abnormal conduction or to the appearance of aberrant morphology in cases of inapparent or subtle preexcitation.

**TABLE 2:** Value of extra‐cardiac vagal stimulation (ECVS) for diagnosing and ablation conduring programmed pacing in common supraventricular tachycardias.

Pacing test	Arrhythmia origin	ECVS	
		Preablation	Postablation
Atrial Stimulation (AS)	AVNRT	AVB	100% AVB
	AVRT (non‐Mahaim)	AVB	100% AVB
	WPW	Aberrancy	100% AVB
	Mahaim	Aberrancy	100% AVB
	Coumel	AVB	100% AVB
Ventricular Stimulation (VP)	AVNRT	99.2% VA block^a^	99.2% VA block^a^
	AVRT (non‐Mahaim)	100% VA conduction	100% VA block
	WPW	100% VA conduction	100% VA block
	Mahaim	100% VA block	100% VA block
	Coumel	100% VA conduction	100% VA block

*Note*: The table summarizes the electrophysiological responses elicited by ECVS during atrial stimulation (AS) and ventricular stimulation (VP) in patients with distinct arrhythmia substrates before and after catheter ablation. Before ablation, ECVS reproducibly induced high‐grade atrioventricular block (AVB) in nodal‐dependent tachycardias (AVNRT, non‐Mahaim AVRT, Coumel) and produced aberrant conduction in accessory‐pathway‐related tachycardias (manifest WPW and Mahaim), thereby unmasking their mechanism. After successful ablation, ECVS provoked complete AVB or ventriculo‐atrial (VA) block in 100% of cases across all substrates, confirming elimination of the re‐entrant pathway and of nodal participation.

Abbreviations: AS, atrial stimulation; AVB, atrioventricular block; AVNRT, atrioventricular nodal re‐entrant tachycardia; AVRT, atrioventricular re‐entrant tachycardia; Coumel, permanent form of junctional reciprocating tachycardia; VA, ventriculo‐atrial; VP, ventricular stimulation; WPW, Wolff–Parkinson–White syndrome.

^a^ In three AVNRT patients, VA block was absent because previous unsuccessful ablation had already produced functional vagal denervation.

In the third test, ECVS was performed during VP to analyze the VA conduction, and it was observed third, high degree, or second degree VA block in 378 of 381 cases of AVNRT (99.2%) (Figure 1G). However, in three cases (0.8%), VA block was not achieved because of prior AV nodal denervation resulting from unsuccessful attempts at AVNRT ablation. Therefore, ECVS induces VA block in all cases of AVNRT, as there is no prior denervation (Table 2).

In the 230 AP patients, ECVS combined with VP demonstrated persistence of VA conduction before ablation in non‐Mahaim cases, 227 (98.6%) of 230 cases. Three patients presented Mahaim fiber and VA block during VP. However, the AP of these cases was previously diagnosed during the test with ECVS and atrial pacing, showing preexcitation under vagal‐induced anterograde AV nodal block, Figure 7A. Postablation, 35 (15.2%) patients exhibited VA block during VP, indicating the absence of retrograde conduction. However, in the 195 (84.7%) patients who still had VA conduction, ECVS combined with VP resulted in VA block in all cases, confirming that the successful ablation of the AP led to VA conduction exclusively via the AV node. After ablation, independent ECVS combined with atrial and ventricular pacing was repeated in all patients, Table 2. Ablation was successful and performed without unusual difficulties in all cases, Table 1. All patients in this study underwent ECVS; therefore, direct comparison of outcomes with and without ECVS was not part of the study design.

### 5.1 Vagal stimulation to revert tachycardia

**FIGURE 5:**
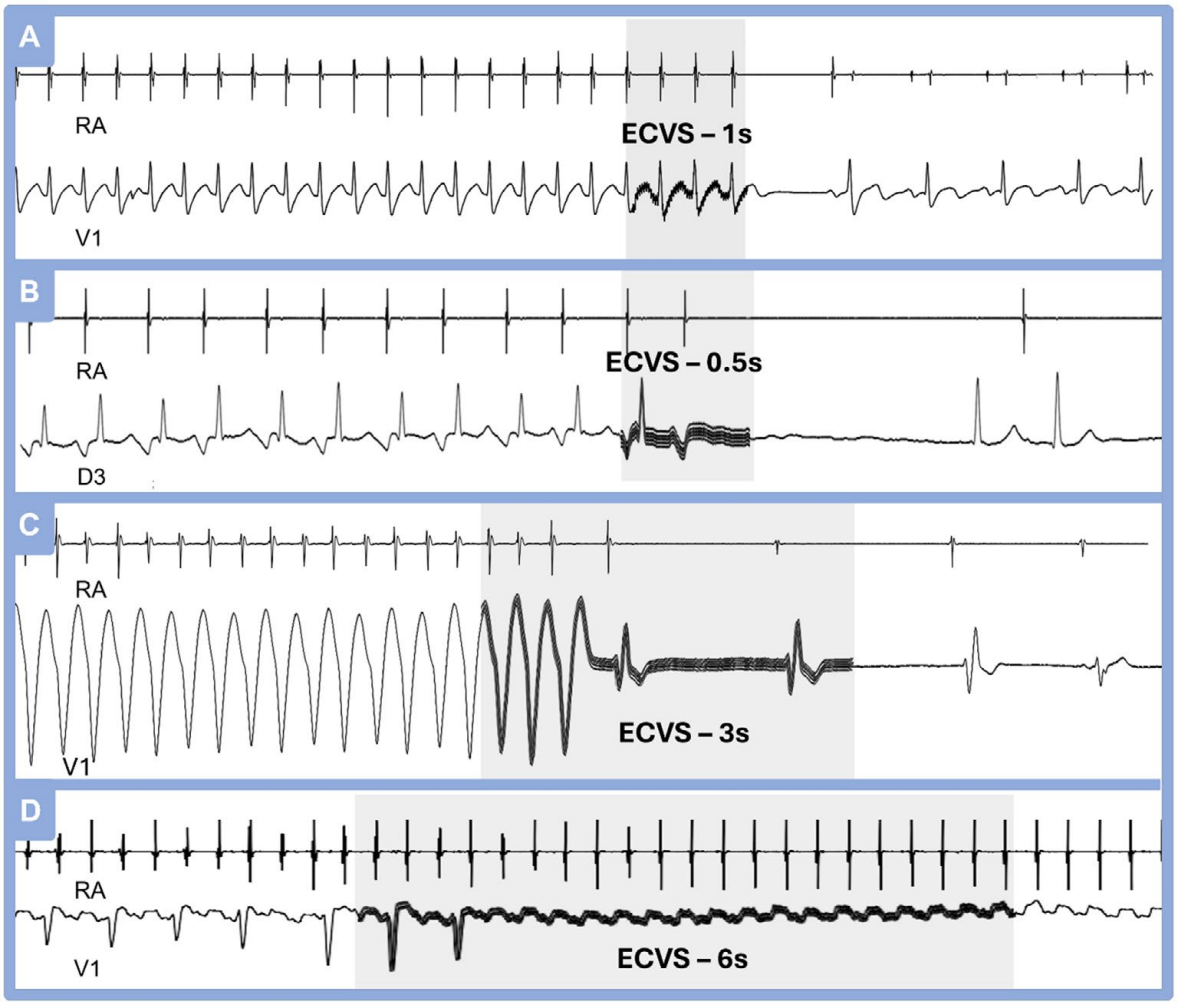
(A) Example of typical nodal reentrant tachycardia induced during an electrophysiological study, quickly reverted with just 1 s of ECVS application. This maneuver is quite helpful since, in some cases, during ablation procedures, there are various spurious inductions of tachycardia. (B) Example of atypical AVNRT reverted with a short ECVS of 0.5 s. (C) Wide QRS nodal reentrant tachycardia reverted with 3 s of ECVS. (D) Different from AVNRT and AVRT, atrial tachycardia or atrial flutter not dependent on the AV node, instead of reversion, typically shows a long period of high‐degree AV block.

Accidental or intentional tachycardia induction can occur frequently during SVT ablation, requiring frequent reversions. In such situations, a short ECVS of 1/2 to 2 s usually reverts the tachycardia, Figure 5A–C proving to be a useful maneuver. The reversion is highly reproducible and equally efficient in AVNRT, typical or atypical, or AVRT tachycardias. In Figure 5C, the rapid reversion of this wide QRS tachycardia by ECVS is another practical tool for validating its supraventricular origin. In Figure 5D, we see an example of atrial tachycardia. Typically, in these cases, ECVS induces a high‐degree or variable AV block without terminating the tachycardia. Despite this, ECVS remains highly valuable as it reveals the P‐wave morphology during tachycardia and, most importantly, confirms that the AV node is not involved, indicating that the tachycardia is solely atrial‐dependent.

### 5.2 Vagal stimulation in Wolff‐Parkinson‐White syndrome

**FIGURE 6:**
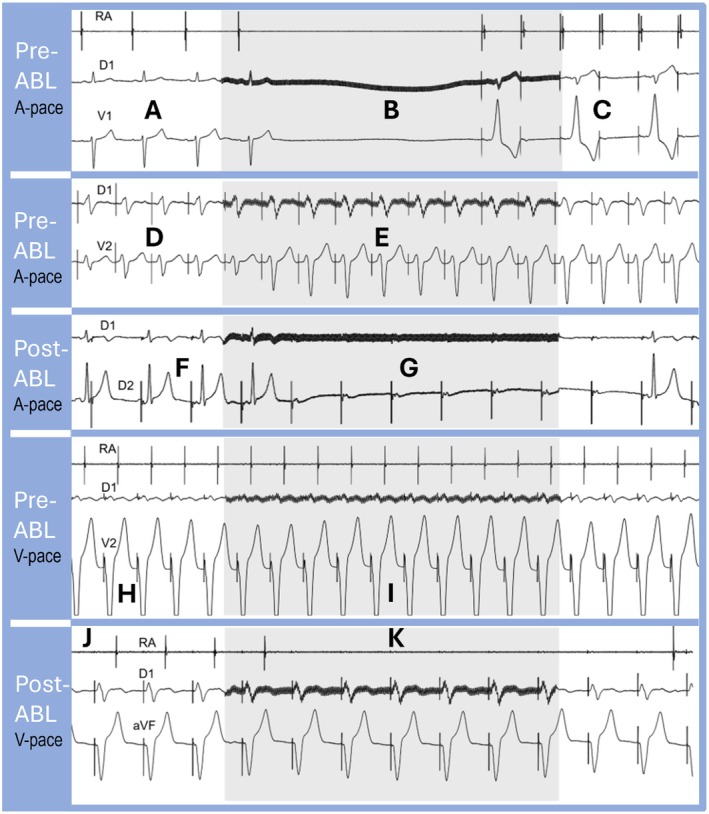
ECVS in two patients with APs. Shaded areas represent the ECVS. (A) Patient 1, with no delta wave despite having a history of intermittent WPW. (B) Sinus arrest induced by vagal effect. (C) Atrial pacing showing 2:1 AV conduction via the AP revealed by the AV node block. In these cases, ECVS commonly reveals the anomalous pathway by blocking the AV node and allowing the alternative abnormal passage (D) Patient 2, with manifest AP (WPW) preablation during atrial stimulation. (E) The ECVS shows an increase in QRS aberrancy, observed because of AV node block with ventricular activation via AP. (F) Patient 2 postablation with atrial pacing, without delta wave and with a narrow QRS. (G) During ECVS, high‐degree AV block is observed because of vagal AV node block. This maneuver is very important to show that the anterograde anomalous conduction of the AP has been completely eliminated and there are no remaining active abnormal bundles. (H) Patient 2 preablation during VP showing 1:1 VA conduction. (I) The ECVS shows persistence of 1:1 VA conduction resistant to vagal action. The lack of VA block during ECVS denotes the presence of anomalous VA conduction. (J) Patient 2 postablation during VP showing 1:1 VA conduction. (K) ECVS causes VA block because of vagal AV node inhibition, demonstrating that the anomalous retrograde conduction has been completely eliminated. Similar to classical EP maneuvers, this behavior shows that before ablation, this patient had dual VA conduction, via the AV node and the AP.

**FIGURE 7:**
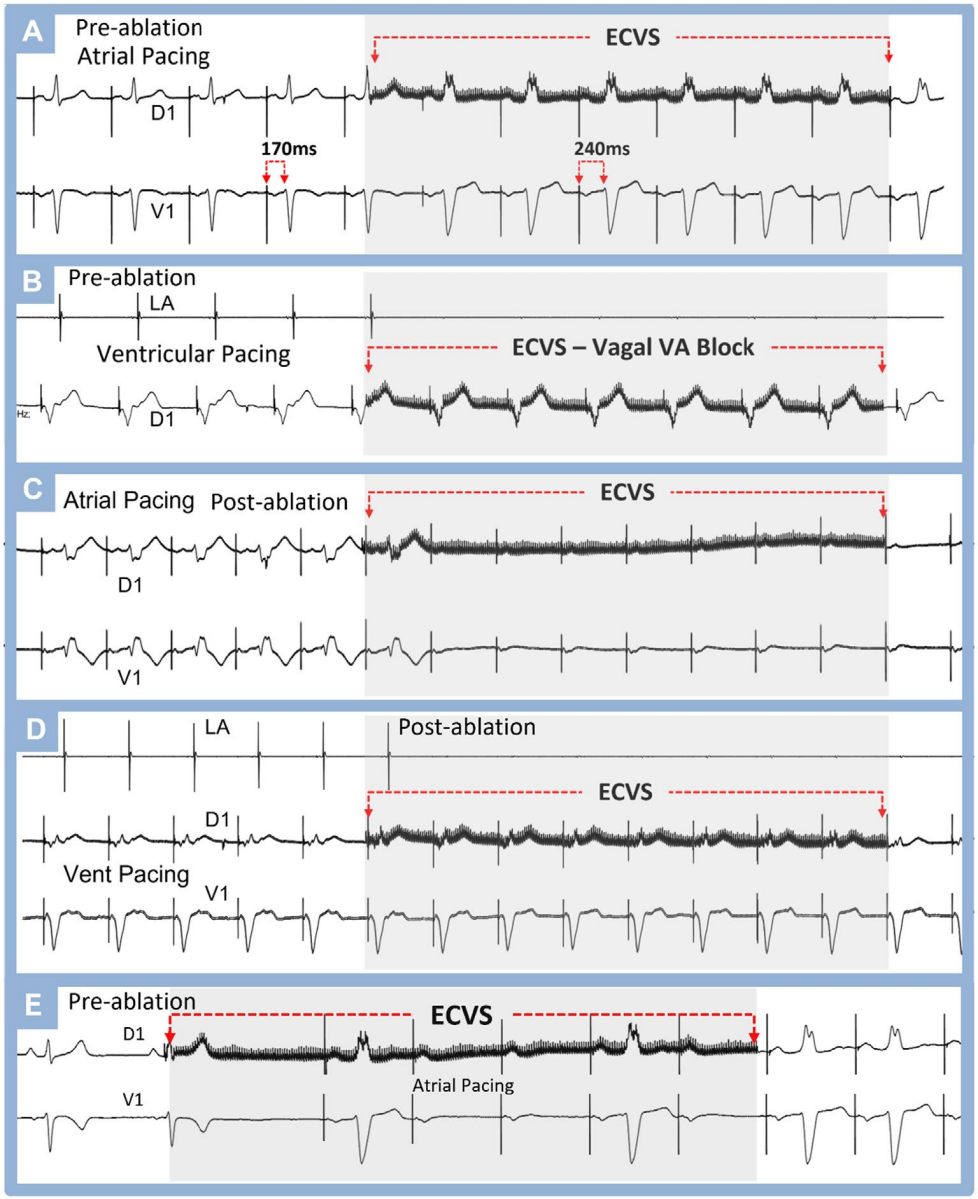
ECVS in a patient with Mahaim fiber tachycardia. (A) Preablation: Atrial pacing demonstrating an AV interval of 170 ms. During ECVS, first‐degree AV block is observed (AV = 240 ms) with QRS widening displaying a complete left bundle branch block morphology. This is attributed to AV node block by the vagal effect of ECVS, which predominantly affects the AV node more than the Mahaim fiber, which maintains ectopic anterograde conduction. (B) Preablation: Right ventricular pacing shows 1:1 VA conduction via the AV node, which is completely abolished by ECVS. (C) Atrial pacing postablation: Normal AV conduction solely through the AV node. ECVS plus atrial pacing demonstrates total AV block following the elimination of the Mahaim fiber. (D) Right ventricular pacing postablation: Normal VA conduction through the AV node, completely blocked by the vagal effect induced by ECVS. This patient exhibited complete right bundle branch block which was not visible on the ECG as the Mahaim fiber masked it during the preablation phase. (E) Preablation showing possible sensitivity of the Mahaim fiber to acetylcholine. ECVS showing complete anterograde block of the AV node and intermittent AV block via the Mahaim fiber. The AV nodal block is more intense and prolonged, leaving AV conduction only through the Mahaim fiber.

In 135 cases of manifest AP (WPW), ECVS associated with atrial pacing (Figure 6B,D) increased the AP manifestation, inducing maximum intermittent or permanent AV conduction through the AP (Figure 6C,E) because of AV node inhibition. This was particularly useful in subtle (Figure 6A), intermittent manifest AP (Figure 6C), or in cases with Mahaim fiber (Figure 7A).

The ECVS protocol (1, baseline; 2, ECVS during atrial pacing; 3, ECVS during VP) was performed at the beginning, and steps 2 and 3 were repeated during and at the end of ablation (Table 2). In 48 (35.5%) patients, an absent or minor (subtle) preexcitation pattern on the resting ECG was observed, (Figure 6A) (intermittent or subclinical/subtle manifest AP) quickly revealed by atrial pacing combined with ECVS (Figure 6C). Aberrancy maximization was the rule in all manifest AP (WPW) patients with ECVS during atrial pacing.

After ablation, ECVS combined with atrial pacing showed transitory high degree or second‐degree AV block (Figure 6G) confirming the elimination and excluding any remaining APs. For comparison, the adenosine test (18 mg) was performed postablation in 89 patients,^17^ resulting in AV block in 85 (95.5%) while second or third degree AV block was observed in all cases with ECVS (100%).

Retrograde conduction was also studied by VP pre‐ and postablation, Table 2. Before ablation, there was VA conduction in all 230 AP patients (Figure 6H). ECVS combined with VP showed VA conduction persistence in 227 of 230 (98.6%) non‐Mahaim cases (Figure 6I). However, in 3 Mahaim cases, there was VA block induced by ECVS (Figure 6B).

A shift in VA conduction (Figure 4B1,B2) was observed in 104 (78.8%) patients, indicating VA block in the AV node, with remaining conduction over an AP. After ablation, only 112 (48.7%) patients persisted with retrograde conduction (Figure 6J). This latter group was subjected to ECVS combined with VP, and first, second, or third degree VA block was observed (Figure 6K), confirming the absence of any remaining AP.

### 5.3 Vagal stimulation in Mahaim fiber

Anomalous ventricular activation via the Mahaim fiber was diagnosed in 3 patients. In Figure 7A, during ECVS with atrial pacing, AV node depression leads to AV interval prolongation and QRS widening, indicating predominant anomalous AV conduction. This behavior helps reveal the presence of the Mahaim fiber. In the three patients, ECVS demonstrated vagal block of VA conduction (Figure 7B,D) and revealed anomalous anterograde conduction during atrial pacing (Figure 7A). In two patients, transient vagal block of the Mahaim fiber was observed, suggesting the presence of some vagal innervation and cholinergic sensitivity (Figure 7E).

### 5.4 Vagal stimulation in atypical AVNRT versus concealed septal AP

By using classical maneuvers to study concentric long VA conduction (Para‐Hissian pace, entrainment and postpacing interval during tachycardia and His synchronous ventricular extrastimulus during SVT) we found 33 (36.7%) patients with atypical AV node reentry (aAVNRT) and 57 (63.3%) patients with para‐septal concealed AP (PSAP), Figures 3B and 5B. ECVS during VP in the 33 patients with aAVNRT resulted in VA block, Figure 3B in 32 patients (97%), suggesting VA conduction only via the AV node. However, in one case (3%), VA conduction persisted during ECVS. This patient had a previous difficult and wide AVNRT ablation that probably caused AV nodal denervation. Interestingly, an adenosine test (18 mg) during VP in this case also showed persistent VA conduction.

In the 57 patients with concealed para‐septal AP, ECVS during VP showed maintenance of VA conduction in all, confirming the presence of an AP and VA block after ablation. There was a match between the EP diagnosis and ECVS; however, ECVS is fast, reliable, and practical: if there is VA block, the diagnosis is AVNRT; if there is persistence of VA conduction during ECVS with VP, there is a very high chance of an AP. Nevertheless, very rare cases of vagal‐resistant VA concentric conduction may be because of AV node denervation.

### 5.5 Vagal stimulation in PJRT

In 3 patients, the clinical presentation was a PJRT. The ECVS demonstrated AV block with atrial pacing and VA block with VP, leading all 3 cases to be classified as atypical AVNRT.

## 6 SAFETY OF VAGAL STIMULATION

No complications related to ECVS were observed. Vascular access through the superior vena cava and internal jugular vein up to the jugular foramen was generally easy. However, some cases presented challenges because of anatomical variations. Difficult access was resolved by utilizing long guidewires, (that must be removed before vagal stimulation once the EP lead is in place). At the conclusion of the procedure and during follow‐up, physical examinations of the neck and jugular vein revealed no issues or discomfort. A direct questionnaire regarding any symptoms related to ECVS was administered immediately postprocedure, the following day, and during follow‐up; no problems were identified. Additionally, no ECG abnormalities associated with ECVS were detected after the procedure or during follow‐up assessments. Despite repeated vagal stimulation, no cases of hypotension were observed. Therefore, the use of vasopressors such as phenylephrine or catecholamines was not necessary.

## 7 DISCUSSION

Modern electrophysiology provides numerous well‐established maneuvers for studying the pathophysiology of SVTs and identifying ablation targets.^2^, ^18^, ^19^ However, this article does not aim to discuss these widely recognized techniques. Instead, it highlights the diagnostic potential of an additional tool, ECVS, that can be incorporated into the electrophysiologist's diagnostic arsenal at their discretion. As a result of the wide spectrum of supraventricular tachycardias evaluated in this large cohort, this study intentionally includes multiple diagnostic scenarios to demonstrate the broad applicability of ECVS.

Control of the parasympathetic effects on the heart, for therapeutic and diagnostic purposes known as vagal maneuvers,^20^ is highly useful. ECVS may achieve this long‐standing objective by allowing controlled local acetylcholine release, which has diverse effects in three primary domains: the sinus node (leading to sinus arrest or sinus bradycardia), the atrioventricular node (resulting in 2nd or 3rd degree AV block), and the atrial wall (causing a reduction in atrial refractory period, atrial refractory dispersion, and AF induction).^21^

This article does not focus on the effects on the atrial and ventricular walls but rather on the impact on AV node conduction. ECVS can act similarly to adenosine, although it targets different receptors. However, neither mechanical stimulation by baroreceptors (vagal maneuvers) nor drugs can match the efficiency, reproducibility, strength, and transience of ECVS. In most patients, ECVS is an easy and powerful technique for precise control over the vagal effects on the heart, and it can be repeated as often as needed in a short time during EP procedures. While ECVS has many potential applications, like in cardioneuroablation^7^ or AF ablation,^21^ this study specifically demonstrates the reproducibility, utility, and safety of this tool in patients with SVT undergoing catheter ablation. The ability of ECVS to be repeated as needed because of the rapid degradation and resynthesis of acetylcholine is particularly valuable. A notable example of acetylcholine's innocuous, transient, and repetitive action arises from skeletal muscle, where the tone depends on cholinergic action 5 to 15 times per second without leaving metabolic or functional residues. This is not possible with another chemical like adenosine, for example.

Subtle noncardioinhibitory vagal stimulation has been studied in heart failure, attempting to harness the potential protective effect of parasympathetic stimulation.^22^, ^23^, ^24^ In contrast, ECVS aims to achieve a transitory and strong cardioinhibitory action. Furthermore, partial vagal effects can also be obtained from high‐frequency stimulation in the atrial endocardium.^25^ However, the response is localized rather than widespread, making this approach time‐consuming and nonspecific. It also easily induces atrial fibrillation, making it neither feasible nor reliable for this application.

Undoubtedly, the transient and controlled induction of nodal AV and VA blocks during the EP procedure for SVT is highly desirable. It offers immediate diagnostic ease and spares numerous time‐consuming maneuvers that could yield indirect or less precise results. ECVS is a straightforward way to leverage the natural model to assist electrophysiologists. Although ECVS was effective across all age groups with no difference in diagnostic capability, longer vagal‐induced pauses were occasionally observed in older patients, likely reflecting age‐related autonomic changes.

Throughout the study, there was no discordance between ECVS and the classical EP maneuvers. ECVS demonstrated perfect concordance with the traditional methods, confirming its accuracy and reliability as an additional diagnostic tool in the management of SVTs.

In this study, ECVS demonstrated several benefits, such as:

1. The induction of sinus bradycardia reveals latent APs, especially those hidden by phase III block.
2. AV node block maximizes the electrocardiographic evidence of APs, allowing for the visualization of subtle (hidden) or intermittent manifest AP (WPW).
3. After ablation, the AV and VA blocks induced by ECVS are highly useful for confirming the elimination of APs and verifying the absence of residual APs.
4. Bidirectional AV node block is a powerful diagnostic tool, especially in challenging cases, to be used at the electrophysiologist's discretion;
5. During EP studies, ECVS allows for the rapid termination of SVT without the electrophysiological stress of other maneuvers.

## 8 LIMITATIONS

Some limitations must be addressed. Patient Selection Bias: 625 patients from a single center were included with standard indications for SVT ablation, focusing mainly on AVNRT and AVRT.

Technical Challenges: Although ECVS was successful in 611 patients (98%), the procedure was not completed in 14 patients (2%) because of difficulties in catheterizing the right or left jugular veins or for not achieving the expected vagal response. This highlights a potential limitation in the universal applicability of ECVS, particularly in patients with challenging vascular anatomy. In these cases, the use of long guidewires for catheterization and ultrasound for localization of the vagus nerve in the neck is highly desirable.

The vagal stimulator may be a potential limitation; however, any neurologic stimulator may be used if adjusted with ECVS‐specific parameters.

Previous vagal denervation: As previously commented, vagal denervation resulting from previous AV nodal area ablations, cardioneuroablation, or because of neural or metabolic pathologies should be considered to avoid false negative results. This aspect is relatively easy to identify and warrants attention to avoid misinterpretations.

While ECVS shows promise as an adjunct tool in diagnosing and treating SVTs, these limitations highlight the need for further research to optimize the technique, expand its applicability, and confirm its long‐term benefits.

## 9 CONCLUSION

Vagal stimulation is an easy‐to‐perform, fast, safe, and reliable additional test for studying AV/VA conduction, diagnosing SVT mechanisms, and assisting in the ablation endpoint. It can be repeated as many times as necessary during EP procedures without complications.

## FUNDING INFORMATION

This research was entirely developed with the authors' own resources and received no specific grant from public or private funding agencies, commercial or not‐for‐profit organizations, or any other sources.

## CONFLICT OF INTEREST STATEMENT

The authors have no conflict of interest to declare.

## ETHICS STATEMENT

This study was approved by the Institutional Review Board of Sao Paulo Heart Hospital.

## PATIENT CONSENT STATEMENT

Informed consent was obtained from all individual participants included in the study.

## Data Availability

The datasets generated and analyzed during the current study are available from the corresponding author upon reasonable request.
